
JOURNAL INFORMATION
==============================
NLM Title Abbreviation: J Comp Med Vet Arch
NLM Journal ID: 17840220R

The Journal of Comparative Medicine and Veterinary Archives

Publisher: Biomedical Journal Digitization Project

ARTICLE INFORMATION
==============================
PMCID: PMC9342565
PMID: 36393502
Article version: 1
Subjects: Points

Points

Print publication date: 1898 Aug
Volume: 19
Issue: 8
First page: 582
Last page: 582
License: This work is free of known copyright restrictions. For more information, please see PMC Back Issue Digitization.
License URL: https://creativecommons.org/publicdomain/mark/1.0/

==============================

Dr. J. C. McNeill, of Pittsburg, Pa., was a visitor to Philadelphia in July and a sojourner at Atlantic City for a short stay.

Dr. A. L. Baum, of Telford, Pa., was a visitor at the Veterinary Department of the Univercity of Pennsylvania in July.

### POINTS.

The granting by the Government of large quantities of black-leg vaccine indiscriminately to stockmen, we learn, has been discontinued, and its dangers and injustice to veterinarians fully appreciated.

A yearling ram of the Lincoln breed recently sold for $5000 and fifty-two yearling rams averaged $435 each. Surely an astounding value in sheep, and points the way to broader instruction in our colleges that will afford a better knowledge in ovine pathology.

Over 500,000 head of cattle have been treated with the Pasteur vaccine for "black-leg" during the past two years, and the estimated saving made is said to amount to five millions of dollars.

A case of docking is running through the Pittsburg courts, inaugurated by the Western Humane Society. The removal of the tails was done with the docking-knife and searing-iron by employés of the person charged with the act. Drs. Rectenwald, Waugh, Carter, and Richards were called as veterinarians for the Humane Society to testify as to the cruelty of the operation done in this manner.

Veterinarians of Kansas are doing much successful work in vaccination for symptomatic anthrax (black-leg) and for malignant anthrax, with very gratifying results.

**PRACTICE FOR SALE.**

A well established $3,500 practice for sale, in a town of 20,000 inhabitants, with several stock farms and good dairy and farming country surrounding. The only qualified Veterinarian in the county. Reason for selling, ill health. This practice is located in Pennsylvania. Would like to sell to some one who would take possession immediately. Address

"M," care of Journal Office,
3452 Ludlow St., Philadelphia, Pa.
